# Case report: Incidentally discovered case of pheochromocytoma as a cause of long COVID-19 syndrome

**DOI:** 10.3389/fendo.2022.967995

**Published:** 2022-09-27

**Authors:** Christian G. Ziegler, Carina Riediger, Matthias Gruber, Carola Kunath, Martin Ullrich, Jens Pietzsch, Svenja Nölting, Timo Siepmann, Stefan R. Bornstein, Hanna Remde, Georgiana Constantinescu

**Affiliations:** ^1^ Department of Molecular Endocrinology and Diabetology, Internal Medicine III, University Hospital Carl Gustav Carus, Technische Universität Dresden, Dresden, Germany; ^2^ Department of Visceral Surgery, University Hospital Carl Gustav Carus, Technische Universität Dresden, Dresden, Germany; ^3^ Department of Endocrinology, Internal Medicine III, University Hospital Carl Gustav Carus, Technische Universität Dresden, Dresden, Germany; ^4^ Helmholtz-Zentrum Dresden-Rossendorf, Institute of Radiopharmaceutical Cancer Research, Department of Radiopharmaceutical and Chemical Biology, Dresden, Germany; ^5^ Technische Universität Dresden (TU) Dresden, School of Science, Faculty of Chemistry and Food Chemistry, Dresden, Germany; ^6^ Department of Internal Medicine IV, University Hospital, Ludwig Maximilian University of Munich, Munich, Germany; ^7^ Department of Endocrinology, Diabetology and Clinical Nutrition, University Hospital Zurich (USZ) and University of Zurich (UZH), Zurich, Switzerland; ^8^ Department of Neurology, University Hospital Carl Gustav Carus, Technische Universität Dresden, Dresden, Germany; ^9^ Division of Endocrinology and Diabetology, Department of Internal Medicine I, University Hospital, University of Würzburg, Würzburg, Germany

**Keywords:** pheochromocytoma (PCC), post COVID-19, plasma metanephrines, complications, diagnostic delay, severe acute respiratory syndrome coronavirus 2

## Abstract

Pheochromocytomas (PCCs) are rare but potentially lethal tumors that arise from the adrenal medulla. The clinical suspicion and diagnosis of PCC can be challenging due to the non-specific nature of signs and symptoms. In many patients, infection with severe acute respiratory syndrome coronavirus 2 (SARS-CoV-2) could lead to long-term symptoms including fatigue, headaches, and cognitive dysfunction. Here, we present the case of a patient incidentally diagnosed with an adrenal mass that proved to be a PCC after imaging was performed due to persisting complaints after coronavirus disease 2019 (COVID-19) infection. A 37-year-old male patient was referred to our center because of a right-sided inhomogeneous adrenal mass, incidentally found during a computed tomographic scan of the thorax performed due to cough and dyspnea that persisted after COVID-19 infection. Other complaints that were present prior to COVID-19 infection included profuse sweating, dizziness, exhaustion with chronic fatigue, and concentration difficulties. The patient had no history of hypertension, his blood pressure was normal, and the 24-h ambulatory blood pressure monitoring confirmed normotension but with the absence of nocturnal dipping. Plasma normetanephrine was 5.7-fold above the upper limit (UL) of reference intervals (738 pg/ml, UL = 129 pg/ml), whereas plasma metanephrine and methoxytyramine were normal at 30 pg/ml (UL = 84 pg/ml) and <4 pg/ml (UL = 16 pg/ml), respectively. Preoperative preparation with phenoxybenzamine was initiated, and a 4-cm tumor was surgically resected. Profuse sweating as well as dizziness was resolved after adrenalectomy pointing toward PCC and not COVID-19-associated patient concerns. Altogether, this case illustrates the difficulties in recognizing the possibility of PCC due to the non-specific nature of signs and symptoms of the tumor, which in this case did not include hypertension and coincided with some of the symptoms of long COVID-19.

## Introduction

Pheochromocytomas (PCCs) are rare chromaffin cell tumors that invariably produce but do not always secrete catecholamines in amounts sufficient to determine hypertension (1). Although intratumoral catecholamine metabolism to metanephrines occurs constantly, catecholamine secretion can be episodic or occur at low rates. Consequently, the clinical presentation of the tumors is highly variable and includes signs and symptoms that are non-specific in nature (2). Thus, among patients tested for PCC, there is also a considerable overlap in the signs and symptoms in patients with and without tumors (3). Furthermore, some of the signs and symptoms of PCC can overlap with those of patients with a positive history of coronavirus disease 2019 (COVID-19) who further develop long COVID-19, post-acute COVID-19, and chronic post-COVID-19 syndrome (4, 5). The term long COVID-19 is usually used to describe any clinical signs, which either persist or newly develop after the acute phase of the COVID-19 infection, last longer than 12 weeks, and cannot be explained by any other diagnosis. Long COVID-19 could manifest with a composite of symptoms that often overlap, fluctuate, or change over time with the potential impact on any infected system of the human body. Furthermore, the number of symptoms associated with long COVID-19 is vast and includes fatigue, muscle and joint pain, headache, insomnia, respiratory problems, heart palpitations, gastrointestinal problems, nausea, dizziness, and seizures. However, the most common complaints in patients with long COVID-19 are fatigue, persistent dyspnea, and neurocognitive disturbances (6). These neuropsychological symptoms are consistent with depression. COVID-19 has also been implicated in evoking changes in the function of the autonomic nervous system, which in recovered patients comprises increased sympathetic nerve activity, including exaggerated sympathetic response to orthostatic stress (7). Such changes may contribute to some signs and symptoms that overlap with those of patients with PCC.

In addition, both patients with PCC and COVID-19 infection have an additional risk for severe disease evolution, since increased catecholamines can promote cardiovascular instability, metabolic dysregulation, and immunologic alterations (5, 8). As a consequence of the aforementioned links between autonomic nervous system function, COVID-19, and catecholamine-related signs and symptoms of PCC, there is potential for confusion in the clinical presentation of both disorders (9). The patient presented in our report provides such an example where the clinical presentation of apparent chronic post-COVID-19 syndrome obscured the clinical suspicion of a PCC but in whom the imaging studies performed because of COVID-19 nevertheless revealed the tumor.

## Case description and diagnostic assessment

**Figure 1:**
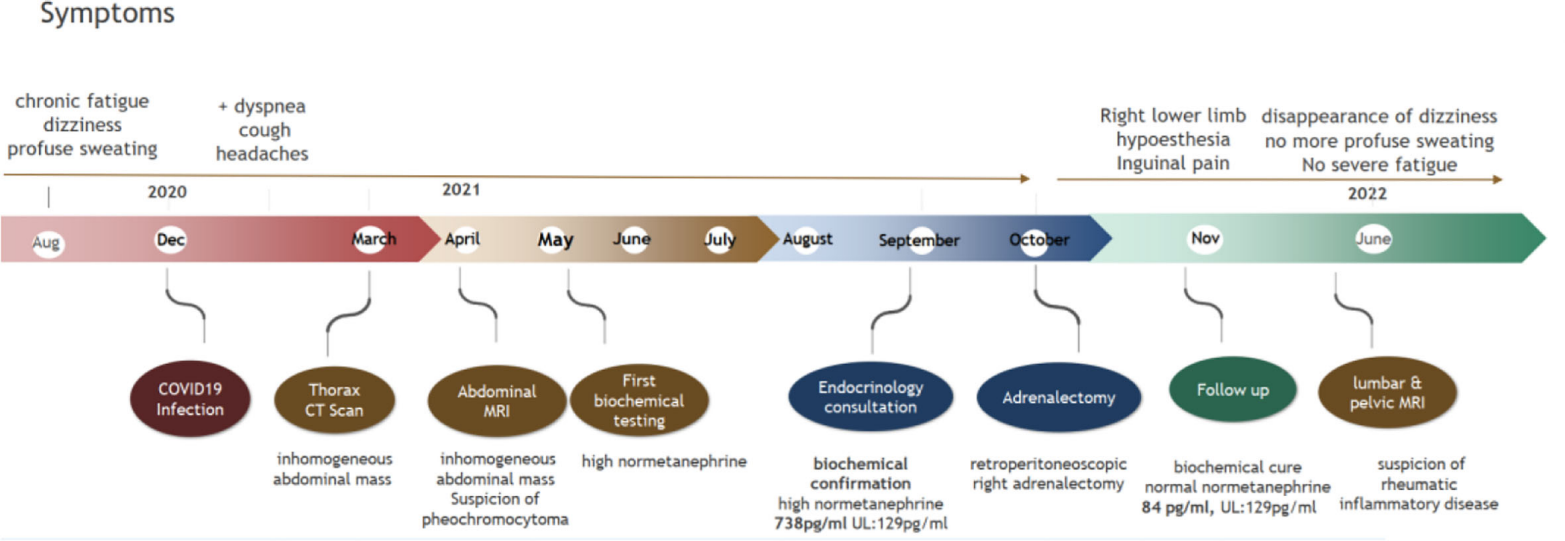
Time course of symptoms and diagnosis.

A 37-year-old male patient was referred to our center by his general practitioner who had incidentally found a 3.8-cm mass in the right adrenal area on computed tomography (CT) scan. The indication for a thoracic CT scan was suspicion of chronic pulmonary lesions or pulmonary embolism after a previous history of COVID-19 infection 3 months prior. Six months before the COVID-19 infection, the patient had complained of dizziness as well as chronic fatigue and profuse sweating with accentuation of symptomology over the summer of 2020 (**Figure 1**).

**Figure 2:**
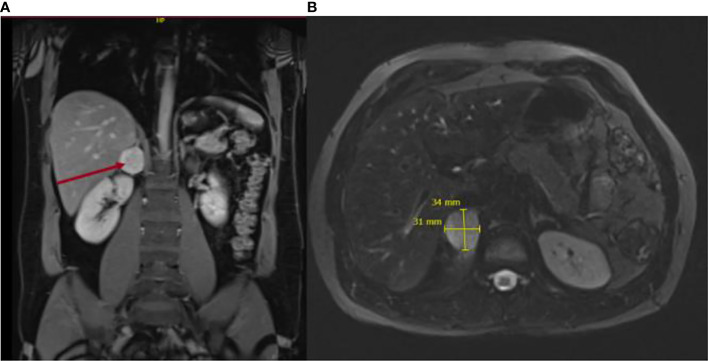
Magnetic resonance imaging depicting axial diffusion-weighted **(A)** and coronal views **(B)** of the 4-cm adrenal mass. .

The patient had no history of hypertension and no previous complaints. In December 2020, he was diagnosed with COVID-19 after presenting with typical symptoms, such as persistent cough, as well as headaches, concentration difficulties, and severe fatigue. He also complained further of profuse sweating and general weakness on a daily basis. He denied any medication or drug intake. On the thoracic CT scan, a right inhomogeneous adrenal mass was identified. An abdominal MRI confirmed the right inhomogeneous mass, presenting no signal loss on out-of-phase imaging, findings that raised the suspicion of a catecholamine-producing tumor (**Figure 2**). Biochemical diagnosis revealed a raised concentration of plasma normetanephrine, and the patient was referred to our endocrine clinic for further investigation.

Clinical examination on referral to our center revealed a well-oriented, afebrile patient with normal body weight (BMI 25 kg/m^2^). His blood pressure was 118/74 mmHg with a pulse rate of 84/min. Cardiac examination revealed rapid but normal heart sounds with no murmurs. Abdominal examination was unremarkable.

A 24-h blood pressure monitoring revealed normal values (122/81 mmHg daytime and 118/75 mmHg over the night). Biochemical evaluation further documented normal renal and thyroid function with normal glycemia. Hepatic enzymes were increased 2-fold. There was no evidence of hypercortisolism (morning cortisol = 402 nmol/L, ACTH 4.3 pmol/L) or primary aldosteronism (aldosterone/renin ratio 39, *N* < 52). The plasma concentration of normetanephrine, at 731 pg/ml, showed a more than 5-fold increase above the age-specific upper cutoff for this metabolite, while metanephrine was 30 pg/ml (*N* < 84 pg/ml), clearly indicating a noradrenergic PCC.

Preoperative preparation with phenoxybenzamine was initiated followed by beta-adrenergic receptor blockade with bisoprolol. Right retroperitoneoscopic adrenalectomy was performed under general anesthesia. The patient showed an uncomplicated postoperative course and was referred to the surgical ward after 24 h of ICU monitoring. Blood pressure was stable and all antihypertensive treatment was terminated.

Histopathological examination confirmed the diagnosis of a 4.0 × 3.6 × 2.8 cm PCC, expressing synaptophysin and chromogranin, S100-positive cells, and SDHB positivity with a mitosis rate of 1/10 HPF/2.4 mm^2^ with the absence of atypical mitoses. The Pheochromocytoma of the Adrenal gland Scaled Score (PASS) was 0, indicating a very low risk of malignancy.

**Figure 3:**
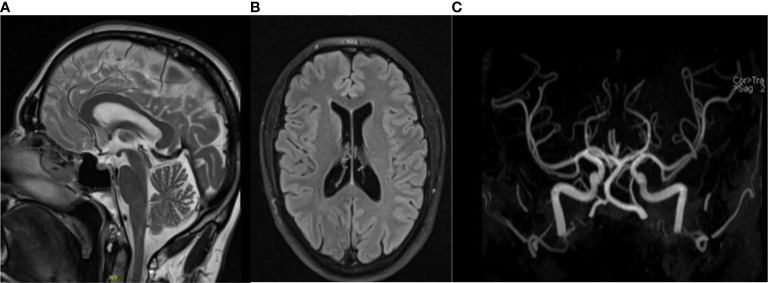
Magnetic resonance imaging depicting T2 flair **(A)** and coronal **(B)** and TOF angiography **(C)** views of the brain, excluding cerebral hemorrhages and stroke.

Postoperatively, the patient complained of right facial paresthesia as well as inguinal pain and hypoesthesia of the ventral side of the right limb and, to a less extent, on the left limb. Neurologic examination could not identify any deficits. Cerebral MRI excluded acute events such as hemorrhage or stroke (**Figure 3**). Completeness of resection was confirmed by normalized results of plasma normetanephrine (84 pg/ml) at 1-month follow-up after surgery. Next-generation sequencing revealed no mutations in over 20 known tumor susceptibility genes, either at the somatic or germline level, including the most prominent genes such as *SDHX*, *RET*, and *VHL*. Postsurgical normalization of plasma normetanephrine, together with the absence of genetic mutation as well as a PASS of 0, led to no further suspicion of metastatic disease. The profuse sweating as well as the dizziness was resolved within the next 6 months after the right adrenalectomy, while the pain, hypoesthesia, and headaches remained. The patient still complained of fatigue, headaches, and loss of concentration within the next months after adrenalectomy but to a more reduced degree than previously experienced. Further neurologic examination showed again no abnormalities aside from hypoesthesia of the ventral side of the right lower limb. The lumbar MRI showed broad-based subligamentous disc protrusion L5–S1 and a slight compression of the sciatic nerve. The inguinal pain was more severe when walking, suggesting degenerative changes in the sacroiliac joint. In the pelvic MRI, there was evidence of a rheumatic inflammatory genesis with slight edema ventrocaudal at the sacroiliac joint.

## Discussion

Some of the most common long-term complications of COVID-19 infection are similar to those reported in patients harboring catecholamine-secreting tumors. Patients with PCC can show well-known symptoms, including the “classical triad” of palpitations, headaches, and excessive sweating. However, various patients present with rather non-specific symptomatology. Due to non-specific signs and symptoms, some characteristics of patients with PCC could overlap with those suffering from post-COVID-19, e.g., chronic fatigue, palpitations, exhaustion, headaches, and dyspnea, as well as anxiety disorders (10). While earlier reports indicated a 50% prevalence of the “classical triad”, a subsequent report by members of our group indicated a lower proportion in the range of 25% (3). Importantly, our patient was completely normotensive, and apart from sweating, not all the other symptoms would certainly arouse suspicion of PCC. While clinicians often put too much emphasis on the presence of hypertension, the absence of a positive history of hypertension in our patient might have contributed to the lack of suspicion of a catecholamine-producing tumor, hindering the timely treatment of this potentially life-threatening tumor (11, 12).

The complications in patients with PCC and COVID-19 are further highlighted by two recent reports. One presented a case of a patient diagnosed with COVID-19 pneumonia and revealed hemorrhage in an incidentally discovered adrenal mass, resulting in a severe catecholamine crisis (12), while another study described an unusual presentation of a PCC as cardiomyopathy complicated by cardiac insufficiency (13). In contrast to the present case, these patients had complications during acute infection. Therefore, prompt and adequate treatment of these patients is of utmost importance to avoid severe outcomes and comorbidities such as stroke, myocardial infarction, circulatory collapse, and death from superimposed risks (8, 9, 11).

Here, we describe the history of a patient who entered our endocrine clinic suffering from suspected long COVID-19 syndrome, without a classical presentation for catecholamine-secreting tumors. This added to the challenges in setting the proper and timely diagnosis of PCC, which is important to avoid life-threatening complications. Finally, a more precise biochemical and imaging analysis proved PCC to be the underlying cause of some of the patients’ long-lasting complaints. Moreover, we show that clinicians should not only focus on classical symptoms such as tachycardia, hypertension, and perspiration, evolving in attacks, since the symptomatology might be less obvious in many patients. Our patient was incidentally diagnosed by imaging performed for symptoms attributed to long COVID.

We acknowledge that one of the limitations in setting the diagnosis was the delay between the first imaging studies raising the suspicion of PCC—first biochemical testing of plasma metanephrines in May and the visit to our clinic in September—confirming the diagnosis that rapidly led to adrenalectomy. The strengths of the study were the multidisciplinary team involved in the diagnostics, the rapid interval from biochemical confirmation to surgery, and the extensive genetic testing and clinical and biochemical follow-up.

As a take-home message of our case, the diagnosis of PCC is often difficult due to non-specific symptoms and, therefore, is frequently delayed. Furthermore, cardinal features like hypertension can be missing. Even in a pandemic situation such as COVID-19, not everything that is unexplained at first glance is long COVID-19, and differential and careful diagnosis should be done under these circumstances, especially in specialized centers of endocrinology.

## Patient prognosis

Since most of the signs and symptoms were resolved after adrenalectomy, the patient’s prognosis seems to be rather good. However, in addition to long COVID-19 fatigue, persistent sacroiliac pain further represents a disability in the patient’s daily life and work performance.

## Data availability statement

The original contributions presented in the study are included in the article/supplementary material. Further inquiries can be directed to the corresponding author.

## Ethics statement

The patient was investigated under an Ethics Committee approved protocol (PROSPHEO- Multicenter PCC and Paraganglioma Evaluation) registered with clinical trials.gov (NCT03344016). The patient provided written informed consent to participate in this study.

## Author contributions

All authors have contributed to the clinical follow-up of the patient and the preparation of this manuscript. All authors contributed to the article, while two authors (MU and JP) also worked on the revised paper version. All authors contributed to the article and approved the submitted version.

## Funding

This work was supported by the Collaborative Research Center Transregio 205 “The Adrenal: Central Relay in Health and Disease” to all listed authors. This study was supported by the Deutsche Forschungsgemeinschaft (314061271-TRR/CRC 205-1/2) to CZ, CK, SRB, HR and GC.

## Conflict of interest

The authors declare that the research was conducted in the absence of any commercial or financial relationships that could be construed as a potential conflict of interest.

## Publisher’s note

All claims expressed in this article are solely those of the authors and do not necessarily represent those of their affiliated organizations, or those of the publisher, the editors and the reviewers. Any product that may be evaluated in this article, or claim that may be made by its manufacturer, is not guaranteed or endorsed by the publisher.

## Abbreviations

PCC, pheochromocytoma; COVID-19, Coronavirus disease 2019; CT, computer tomography; MRI, magnetic resonance imaging.
